# 微生物组学在肺癌发生发展中的作用机制及研究进展

**DOI:** 10.3779/j.issn.1009-3419.2020.101.38

**Published:** 2020-11-20

**Authors:** 国慧 刘, 安鑫 谷, 明艳 鄂

**Affiliations:** 150040 哈尔滨，哈尔滨医科大学附属肿瘤医院放疗科 Department of Radiation Oncology, Harbin Medical University Cancer Hospital, Harbin 150040, China

**Keywords:** 肺肿瘤, 微生物菌群, 作用机制, 气道微生物组, Lung neoplasms, Microbial flora, Mechanism, Airway microbiome

## Abstract

肺癌是我国最常见的、预后较差的恶性肿瘤之一，大多数患者确诊时即为晚期。有研究表明肺癌患者的微生态特征有别于健康人群，呼吸道的微生物可以通过多种机制影响肺癌的发生发展。近年来微生物组学与疾病相关性研究已成为继人类基因组计划又一研究热点，然而目前有关肺癌与呼吸道微生物组特征的研究相对较少，因此，需要更深入地探讨肺癌与微生物菌群间的潜在联系，通过研究呼吸道微生物在肺癌发生发展中的作用机制，以期在为肺癌的临床诊治、预后评估提供更明确的科学依据。本文对目前关于微生物菌群与肺癌相关研究进行综述，并为临床上诊断与治疗肺癌提供新的思路。

## Abstract (en)

Lung cancer is one of the most common malignant tumors with poor prognosis in China, and most patients are diagnosed as advanced patients. Studies have shown that the microecological characteristics of lung cancer patients are different from healthy people, and the microorganisms of the respiratory tract can affect the occurrence and development of lung cancer through various mechanisms. In recent years, the study of the correlation between microbiome and disease has become another research hotspot following the Human Genome Project. However, at present, there are relatively few studies on the characteristics of lung cancer and respiratory microbiome. Therefore, it is necessary to further explore the potential relationship between lung cancer and microbial flora. By studying the mechanism of action of respiratory microorganisms in the development of lung cancer, it is expected to provide a clearer scientific basis in the clinical diagnosis, treatment and prognosis assessment of lung cancer. This article reviews the current researches on microbial flora and lung cancer, and provides new ideas for clinical diagnosis and treatment of lung cancer.

肺癌是我国最常见的恶性肿瘤之一，其死亡率占所有恶性肿瘤之首。肺癌患者早期可无明显症状，当患者出现呼吸道或者肿瘤相关症状时，肿瘤可能已经处于进展期。目前肺癌患者确诊时大多已是局部晚期或出现广泛转移，其5年生存率在20%以下^[1]^，总体预后仍然很差^[2]^。

近年来，随着微生物组学的深入研究与发展，我们对呼吸道病原学的理解有所改变。既往认为肺部是无菌的，但现有的技术可以在正常人的肺泡灌洗液和肺组织中检测到微生物，可能源于口咽部菌群的迁移及空气吸入^[3]^。这些研究结果均证实，下气道不是绝对无菌的，同时数据结果显示特定的微生物和人类微生物谱学差异都可能引起致癌作用，并且可能通过不同的生物学途径对癌症的诊治及预后产生影响^[4]^。

有研究^[5, 6]^显示肺部不仅存在微生物，而且肺内微环境的差异，如氧张力、pH值和免疫状态均可能造成肺部微生物谱的改变。通常情况下体内的微生物菌群可维护人体的健康平衡，但一旦某些因素的作用下出现生态失调，就可能会影响局部微环境，进而影响正常的细胞代谢，导致疾病的易感性增加，最终发生恶性肿瘤。大约20%的肿瘤病因与微生物感染直接关联。人体微生物组可能在促癌、诱癌中发挥调节作用，也可能是肿瘤的真正病因。在Peters等^[7]^的研究中，首次阐述了呼吸道微生态与非小细胞肺癌患者预后间的相关关系。然而目前关于肺癌患者肺部组织微生物菌群的改变在肺癌发生发展中的研究报道相对较少，本文将对肺癌与微生物菌群间的潜在联系，呼吸道微生物在肺癌发生发展中的作用机制加以综述，以期在肺癌的临床诊治、预后评估中提供更明确的科学依据，并为肺癌的预防控制提供新靶点。

## 1 呼吸道微生物菌群概述

相关研究证实，在健康的呼吸道黏膜中存在着相关菌群，其中，细菌密度最高的为上呼吸道，鼻腔和鼻部每鼻拭子可达到10^3^-10^6^个活菌。气管和肺内采集的支气管肺泡灌洗中估计细菌数量约有10^2^个细菌/mL^[8, 9]^。健康的人体下呼吸道中存在着固定的菌群，其在门、纲的水平以变形菌纲、硬壁菌门、梭杆菌纲和拟杆菌门为主，在属的水平以假单胞菌属、链球菌属、普雷沃菌属、韦荣球菌属、嗜血菌属以及奈瑟球菌属为主^[10]^。

呼吸道微生物在生命早期迅速增长，并且受环境、年龄和免疫状况的影响^[11]^。目前证明，出生方式、生后第1 h生活环境和出生后3个月-4个月的生活环境暴露是呼吸道菌群稳定发展的重要阶段^[12]^。而这种微生物菌群与呼吸系统健康和疾病之间存在着动态的平衡，当呼吸道防御受到侵袭时，下呼吸道对微生物的暂时性暴露可能导致肺内复杂微生物菌群的建立，从而引起疾病的发生。

## 2 肺癌与微生物组学

肺癌在肿瘤中发病率居于第一位，约超过100万亿个微生物栖息在人类呼吸道，其中包括健康的人群和癌症患者等。烟草为肺癌发病的重要因素之一，含有的多环芳烃类等致癌物在体内的代谢主要受人体微生物的影响，所以说微生物很可能和肺癌的形成发展是有关联的。肺结核分支杆菌以及幽门螺杆菌感染均能促进肺癌的发生。从肺癌患者下呼吸道中检测出多种厌氧菌，其中最多的是放线菌属和消化链球菌属。Hosgood等^[13]^通过对长期暴露于油烟的非吸烟女性肺癌患者的呼吸道微生物研究证实，患者痰液中的微生物种类明显多于正常对照组。肺癌与呼吸道微生物组之间关系的研究虽然仍处于起步阶段，但越来越多的研究表明呼吸道中微生物在肺癌发生发展中发生了很大变化。

肺微生物群是一种结合微生物、遗传物质和微生物基因产物的综合体。Apopa等^[14]^对肺活检组织的微生态构成进行的研究中发现肺癌患者有相似的微生态构成。微生物菌群包含的微生物种类其实是很多的，但是关于肺微生物群在肺癌的发生作用上目前仍然需进一步探索。目前研究重点需进一步阐明微生物组学在肺癌发生发展中的具体作用，能为肺癌的发病机制以及诊断治疗提供新的证据。

## 3 微生物组学在肺癌发生发展中的作用机制

随着呼吸道微生物组学相关研究的逐步开展，现已意识到肺癌患者气道微生物组的特征与健康人群的呼吸道菌群有特异的结构上的差异，而这种动态的变化可能与恶性肿瘤存在某种相关性^[15, 16]^。最新研究进一步报道了微生物组学在肺癌发生发展的作用，提出了几种可能的作用机制：①菌群微生态失调；②遗传毒性和毒力效应；③代谢合成途径；④炎症反应；⑤免疫反应。

### 3.1 菌群微生态失调

微生物菌群共生于我们的生存环境中，并起到重要的作用，各种微生物与宿主之间形成了一个极为复杂的有机体^[17]^。在呼吸系统中，菌群的微生态平衡能够维持内环境的稳定，同时免疫系统也维持着细菌、真菌与宿主之间的互利共生、共同进化的平衡关系。因此，在维持正常的生理环境和进行正常的生理功能过程中，菌群微生态失调可通过产生过多的毒性物质、介导炎症反应来引起肺部疾病甚至肺癌的发生发展。

一项对照研究结果^[18]^表明，在肺癌患者的痰液中草绿色链球菌、毗邻颗粒链球菌、结核分枝杆菌丰度高于非肺癌患者。Hosgood等^[13]^进行的研究选取了腺癌患者及健康人群为对比，通过检测8例非吸烟的女性肺癌患者及8名非吸烟的健康对照者的痰液标本，发现健康人群颗粒链球菌属、链球菌属等菌属的相对丰度较肺癌患者明显下降，结果提示了室内的燃煤烟雾可能与呼吸道菌群失调发挥协同作用，并对肺癌的发生有促进作用。另一项研究^[19]^对肺癌患者病变部位进行刷检所得的标本进行测序，结果发现链球菌属的相对丰度在肿瘤病变处的肺泡灌洗液中是最高的，其次是病变对侧的肺泡灌洗液，正常健康人群的肺泡灌洗液中链球菌属的相对丰度最低，继而提出“菌群迁移”的概念，并可能与肺癌的发生发展有关联，进而提示，菌群结构的改变参与了肺癌的发生^[20]^。

Yu等^[21]^的研究中发现恶性肿瘤的肺组织中的物种多样性较非恶性肿瘤的肺组织相对较低，从肿瘤的分期角度看，肿瘤原发灶-淋巴结-转移（tumor-node-metastasis, TNM）分期为Ⅲb期及Ⅳ期的晚期肺癌患者的栖热菌属相对丰度明显升高，而伴有远处转移的肺癌患者的健康肺组织中则含有较多的军团菌属，进一步表明部分微生物菌群之间的动态变化可能与肺癌的进展有关。基础实验^[22, 23]^发现肺部副流感嗜血杆菌及肺部菌群失调对小鼠癌细胞转移到肺部有促进作用，这说明肺部菌群参与了肺癌的转移。P53肿瘤蛋白（tumor protein p53, *TP53*）是肺癌最常见的突变基因^[24]^，其突变可以破坏气道上皮屏障，影响肺部菌群，利于细菌的侵入与繁殖。Greathouse等^[25]^研究证实食酸菌属的丰度在*TP53*突变的肺鳞癌组织中高于TP53野生型肺鳞癌。

因此，肺部微生物菌群既可以促进肺癌的发生发展，而宿主因素例如吸烟、基因突变也可以影响肺部微生物菌群状态。在正常机体中存在着一道天然的黏膜保护屏障，可以抵御微生物菌群的感染。一些共生的菌群在一定条件下可导致疾病的发生发展。因此，菌群微生态失调在我们临床诊治上有着重要的作用，而这种致癌的机制需要临床医护人员以及研究者的进一步探索来证实。

### 3.2 遗传毒性和毒力效应

微生物本身及其代谢产物中的内毒素、蛋白酶、纤维蛋白溶酶和脂肪酸等，对宿主细胞均有毒性作用，可以直接诱发细胞癌变，或者间接改变细胞的信号转导通路，对肿瘤的发生起促进作用。微生物感染能够逃离机体免疫系统形成氧自由基、一氧化氮、基质金属蛋白酶等毒力因子，以促进肿瘤的发生。微生物也可通过激活有丝分裂原活化激酶（mitogen-activated protein kinase, MAPK）通路诱导细胞增殖，增加基因突变，提高肿瘤的转移率和发病率。同时，菌群感染引起的胞内累积效应可以通过调节B淋巴细胞瘤-2（B-cell lymphoma-2, Bcl-2）家族蛋白的表达或灭活视网膜母细胞瘤抑制蛋白（retinoblastoma inhibitory protein,pRb）来抑制细胞凋亡，促进癌细胞的转化。

### 3.3 代谢合成途径

肺癌与微生物组学之间的关系正在逐渐成为研究热点，现已发现微生物群落与肺癌的发病率和死亡率之间存在关联。Boyton等^[26]^表明，人体内的微生物组在肺癌的发生发展中起着核心作用。研究发现在肺癌中一些代谢产物如胆碱磷酸、牛磺酸、谷胱甘肽、谷氨酰胺、精氨酸等会发生显著改变。微生物在胆汁酸和蛋白质的代谢中能导致芳香胺和硫化物的形成，以促进肿瘤的生长。

Hosgood等^[13]^对不吸烟女性肺癌患者进行研究，将其痰液微生物菌群组与该地区正常人对比，发现肺癌患者痰液中颗粒链菌属（6.1%）、营养缺陷菌属（1.5%）以及链球菌属（40.1%）的相对丰度明显高于正常人（分别为2%、0.085%和19.8%），并推测呼吸道微生物可能影响了多环芳烃等致癌物在体内的代谢，参与了肺癌的发生。研究证实颗粒链球菌通过参与腐胺及聚胺的代谢途径影响肺癌的发展^[27]^，而腐胺及聚胺的降解可以影响细胞周期^[28]^。Ye等^[29]^中，将手术切除下来的肺癌组织进行原代细胞培养，发现在革兰阴性杆菌刺激培养后Toll样受体（Toll-like receptor, TLR）4及TLR9通路被激活，脂质合成增加，导致肿瘤的进展和转移。体外研究中这些患者的肿瘤细胞也显示出更强的转移能力，提示不当的脂质合成影响肺癌的发生及转移。

### 3.4 炎症反应

肿瘤微环境是癌细胞生存的内部环境^[30]^，癌细胞的过度增殖和异常分化对其他细胞的迁移运动及炎症反应有影响^[31]^。目前感染被认为是诱发肿瘤的主要致癌因素，炎症通过诱导基因组突变、异常性组织修复和增生反应等过程将微生物与癌症的发生联系起来。微生物菌群可通过引发慢性感染或产生细胞毒素，引起DNA损伤，影响细胞周期，是肿瘤发展转移的重要驱动因素。

浸润的中性粒细胞促进肿瘤相关的炎症、血管生成及转移。在甲基胆蒽诱导的肺癌小鼠模型中，中性粒细胞的消耗与肺癌发生显著减少有关^[32]^。Brenner等^[33]^的研究发现肺部结核分枝杆菌感染会增加发生肺癌的风险。Erb-Downward等^[34]^报道肺泡结构的破坏影响部分细菌的生存环境，减少的流感嗜血杆菌会迁移生存到气道。机体对肺部菌群改变的反应性调控会增加免疫细胞，细菌无法适应免疫改变即被淘汰，如黄杆菌属随着CD4^+^T细胞的增加而减少。Jungnickel等^[23]^发现异常细菌诱导的上皮细胞因子白介素-17C抗体（interleukin-17C antibody, IL-17C）通过增加嗜中性粒细胞炎症反应来介导细菌的促癌作用，可见IL-17C具有促进肿瘤相关炎症和肿瘤增殖的功能。

Dickson等^[35]^研究发现小鼠的肺部菌群能反应小鼠的肺部免疫状态，这提示肺部菌群可以通过炎症反应促进肺癌发生发展。在Jungnickel等^[23]^的研究中，注射LCC细胞的小鼠先后暴露在烟草及副流感嗜血杆菌，暴露于副流感嗜血杆菌组的小鼠的肺转移结节数量及体积均较未暴露组增多增大，进一步显示副流感嗜血杆菌通过Toll样受体2/4上调IL-17C的表达，诱导肿瘤周围中性粒细胞的浸润，促进肿瘤发展。基础实验证实肺炎链球菌可通过Toll样受体2上调白介素-6（Interleukin-6, IL-6）的表达，进而促进肺癌细胞转移^[36]^。Gowing^[36]^发现经肺炎链球菌处理后的H59及A549非小细胞肺癌细胞激活Toll样受体2-髓样分化因子88（myeloid differentiation factor88, Myd88）信号通路，促进IL-6的释放，促进非小细胞肺癌的远处转移。另一体外研究^[27]^中发现增多的韦荣球菌通过释放相关炎症因子进一步上调外调节蛋白激酶-磷脂酰肌醇3激酶（extracellular regulated protein kinases-phosphatidylinositol 3 kinase, ERK-PI3K）通路影响肺癌的发展。

总之，目前认为肺癌的形成过程不是仅由几个细菌所决定，更可能是气道微环境与菌群失衡而出现的炎症反应，这种炎症反应的出现与宿主免疫状态、病原体的入侵及治疗干预密切相关。

### 3.5 免疫反应

肿瘤的免疫微环境沉浸于各种免疫细胞中，含有众多的细胞、分子及信号通路^[37]^。目前已知微生物菌群的细胞壁成分和代谢产物都参与调节宿主对微生物和环境刺激的免疫反应，并通过多种机制影响肺癌患者机体的免疫细胞。Liang等^[38]^研究表明，T调节细胞、M2巨噬细胞及激活的中性粒细胞等免疫细胞在肺部微环境中促进肿瘤生长。

具核梭杆菌会产生一种特殊蛋白参与到T细胞和自然杀伤细胞的免疫受体的作用中，阻断免疫细胞对肿瘤细胞的毒性效应。来源于长双歧杆菌的表面多糖可抑制肺部选择性T辅助细胞17型细胞（Helper T cells 17, Th17）反应。Song^[39]^研究进一步表明，微生物诱导的Th17可促进肺癌细胞增殖和血管生成。Tsay等^[27]^证明微生物组可调控癌症免疫应答，小鼠模型中发现产肠毒素脆弱类杆菌（enterotoxigenic bacteroides fragilis, ETBF）以Th17触发信号传导转录激活因子3（signal transducer and activator of transcription 3, STAT3）活化，通过Th17依赖性途径诱导癌症。另一项实验^[7]^表明，小鼠暴露于副流感嗜血杆菌中6个月后，肺部炎症细胞数量增加，通过促进微血管生成及上调缺氧可诱导因子1（hypoxia-inducible factor 1, hif-1a）表达促进小鼠肺部肿瘤的发生。Jin^[40]^研究表明，肺癌小鼠肺部细菌数量的剧增导致了γδT细胞分泌IL-17及IL-23，中性粒细胞所释放的炎症物质共同促进肿瘤的生长，抗感染治疗后小鼠体内的肿瘤体积明显缩小，提示细菌导致了剧烈的免疫反应刺激肿瘤生长，而抗感染治疗具有抗肿瘤效应。

已有研究^[41, 42]^表明接受免疫治疗的非小细胞肺癌患者接受抗感染治疗后其无进展生存期（progression-free survival, PFS）及总生存期（overall survival, OS）均较无抗感染者要差，且菌群与免疫治疗的不良反应相关。Liu等^[43]^研究发现抗生素的应用可能导致拟杆菌属、韦荣球菌属等菌群失衡，增加了接受抗程序性死亡受体1（programmed cell death protein 1, PD-1）抗体治疗的肺癌患者罹患免疫相关性反应的风险。近年来免疫治疗在肺癌中的应用及免疫异常在疾病发病过程中备受关注，肿瘤浸润免疫细胞和免疫检查点对肿瘤的发生、进展和生存预后均发挥着重要的作用^[44]^。这体现了肺癌治疗从以杀瘤为主向以调节肿瘤微环境为主的转变^[45]^，而治疗理念的转变也是肿瘤精准治疗取得临床疗效的关键。

## 4 展望

目前研究已证实肺癌患者的肺部存在许多差异菌属，而肺部微生态也存在改变。近年来，肺癌发病率逐年增加，肺部微生物组学研究正逐渐拉开序幕，其独特的解剖位置、取样的多样性和困难、器官的功能重要性均提示这个领域的研究有待深入研究。现已从微生物组学角度对肺癌病因进行探索，仍需大样本的前瞻性队列研究进一步研究来表明肺癌的发生发展与微生物菌群存在相关性，并通过干预手段实现对菌群变化的监测^[46]^。针对微生物菌群在肺癌发生发展中作用机制的研究将有助于对肺癌的深入认识，明确机制后需进一步延缓或阻止呼吸道肿瘤的发生发展^[47]^。对患者进行微生态的靶向治疗，或是利用微生物菌群作为生物标记物来评估患者的患病风险，病变程度以及预后情况，有利于制定新的临床策略，进而开发一种简单易行、早期发现肿瘤的新方法，以期得到更好的肺癌临床治疗效果。

总之，微生物组学在癌症中扮演着至关重要的角色。我国是肺癌的高发国家，深入研究呼吸道菌群在肺癌发生发展中的作用，能够更好地重新认识微生物菌群与肺癌的关系，从而为肺癌预防和治疗提供新的思路，是未来精准医学的重要方向。
